# Association of age, sex and BMI with the rate of change in tibial cartilage volume: a 10.7-year longitudinal cohort study

**DOI:** 10.1186/s13075-019-2063-z

**Published:** 2019-12-09

**Authors:** Guoqi Cai, Matthew Jiang, Flavia Cicuttini, Graeme Jones

**Affiliations:** 1 Menzies Institute for Medical Research, University of Tasmania, Private Bag 23, Hobart, TAS 7001 Australia; 2 Department of Rheumatology, Royal Hobart Hospital, Hobart, TAS Australia; 3 Department of Epidemiology and Preventive Medicine, Monash University Medical School, Melbourne, Australia

**Keywords:** Age, Cartilage volume, Magnetic resonance imaging, Osteoarthritis

## Abstract

**Background:**

To describe the association of age, sex and body mass index with the rate of change of tibial knee cartilage volume over 10.7 years in a community-based sample of older adults.

**Methods:**

Four hundred and eighty-one participants (49% female, mean age 60.8 years [range 51.1–79.7], 49% had knee pain and 58% radiographic osteoarthritis) were included. Tibial cartilage volume of the right knee was assessed on T1-weighted fat-suppressed 1.5 T MRI at baseline and 10.7 years. Data analyses were performed using linear regression models.

**Results:**

The average rate of loss of cartilage volume was 1.2%/year (range 0.2–3.9%) with all participants losing cartilage volume over the study period. There was a significant association between age and loss of tibial cartilage volume in the medial (0.023%/year, 95% confidence interval [CI] 0.010 to 0.036%, *p* < 0.001), lateral (0.013%/year, 95% CI 0.003 to 0.023%, *p* = 0.012) and total tibia (0.018%/year, 95% CI 0.009 to 0.026%, *p* < 0.001). Higher body mass index at baseline and increases in body mass index over time were associated with a greater tibial cartilage loss at the medial (body mass index at baseline 0.040%/year, 95% CI 0.022 to 0.058%, *p* < 0.001; increases in body mass index 0.055%/year, 95% CI 0.018 to 0.093%, *p* = 0.004) but not lateral compartment. No evidence of non-linear relationships was observed. Compared to males, females lost more lateral tibial cartilage with increasing age (0.023%/year, 95% CI 0.003 to 0.043%, *p* = 0.024 for interaction).

**Conclusions:**

Tibial cartilage volume declines at a faster rate with increasing age and body mass index in both males and females, particularly in the medial compartment. In contrast to the low rate of change in radiographs, our findings suggest that cartilage loss at the tibia is universal in this age group.

## Background

Osteoarthritis (OA) is the most common form of arthritis, characterised by gradual loss of articular cartilage [1]. The prevalence of OA increases with age implying that the disease progresses with age. However, current evidence concerning the role of age on the structural progression of OA is inconsistent.

Radiographic joint space width at the tibiofemoral joint has historically been considered a good measure of change in cartilage volume; however, radiograph-based studies have reported inconsistent findings with regard to joint space or cartilage loss with age [2–7]. While most studies show low rates of progression over time and that this only occurs in some subjects [2, 6, 7], this may reflect inaccuracies with radiographs over time. Change in knee cartilage volume on magnetic resonance imaging (MRI) correlates poorly with the change in radiographic joint space width [8], which suggests that radiographic change may not be sensitive to cartilage loss. MRI-based cartilage loss has been shown to have greater sensitivity to change [9–12] and is recognised as a valid, accurate and reproducible tool to measure articular cartilage volume [13–15] and its rate of change [16–19]. We have previously reported on age and cartilage loss in a younger population suggesting cartilage loss is almost universal after the age of 40 but there are no studies in the elderly [1, 20]. MRI-based studies have also demonstrated age to be associated with increased severity and prevalence of cartilage defects [14] as well as cartilage thinning [21]. Moreover, there is a recognised sex difference, with women having a higher prevalence of OA than men, particularly beyond the age of 50 [22], and there are also sex differences in cartilage loss in middle-aged adults [1]. In older adults, cartilage loss may be more likely to vary between sexes due to changes in hormone levels in postmenopausal women, which are associated with progressive articular structural changes [23]. Moreover, a significant sex difference in growth factors has been confirmed in older adults [24]. Importantly, these growth factors including transforming growth factor-β and insulin-like growth factor-1 play an important role in cartilage formation and repair [25].

Previous population-based studies have evaluated change in cartilage volume over a relatively short time of only 2 years [1, 16–20] showing a deleterious [1, 19, 20] or no association [16–18] between age and greater cartilage loss. The inconsistent results may be explained by the increased cartilage thickness at early stages of OA due to swelling or softening of cartilage [26–29]; a longer follow-up in older adults would minimise such influence. Moreover, high body mass index (BMI) is a major risk factor for the development and progression of OA [30]. However, there is limited evidence showing the effect of BMI on knee cartilage volume, especially with a long-term follow-up [31]. Gersing et al. [32–34] found that weight loss may slow knee cartilage deterioration in overweight and obese individuals over 48 and 96 months, in which cartilage deterioration was assessed using the modified Whole-Organ Magnetic Resonance Imaging Score (WORMS) [32] or knee cartilage T2 values [33, 34]. The aim of this study, therefore, was to describe the association of age, sex and BMI with the rate of loss of tibial knee cartilage volume in a population-based sample followed up over a 10.7-year period, which was much longer than previous studies. Moreover, this population-based cohort consisted of older adults (50–80 years), while previous studies have included only middle-aged adults (< 60 years) [1, 20]. We hypothesised that tibial cartilage loss would be greater with increasing age and BMI, especially in females.

## Patients and methods

### Study participants

The Tasmanian Older Adult Cohort (TASOAC) study is an ongoing prospective study in southern Tasmania that began in 2002 [35]. Men and women (98% Caucasian) aged 50–80 years old were randomly selected from the electoral roll in southern Tasmania (population 229,000), a comprehensive population listing using sex-stratified random sampling without replacement (response rate 57%). Participants were excluded if they were institutionalised or had contraindications to MRI. There were no other participation restrictions. The study was approved by the Southern Tasmanian Health and Medical Research Ethics Committee, and written informed consent was obtained from all participants.

Baseline measurements (phase I) were conducted from April 2002 to September 2004. Follow-up data were collected at 2.7 (range 1.7 to 2.9), 5 years (range 4.6 to 5.9) and 10.7 years (range 9.2 to 12.5). A total of 1099 participants were enrolled in this cohort at baseline, and 569 (51.8%) participated the 10.7-year follow-up. The current study consists of 481 participants who underwent MRI assessments and had validated MRI scans at baseline and the latest (10.7-year) follow-up.

### Magnetic resonance imaging

**Fig. 1:**
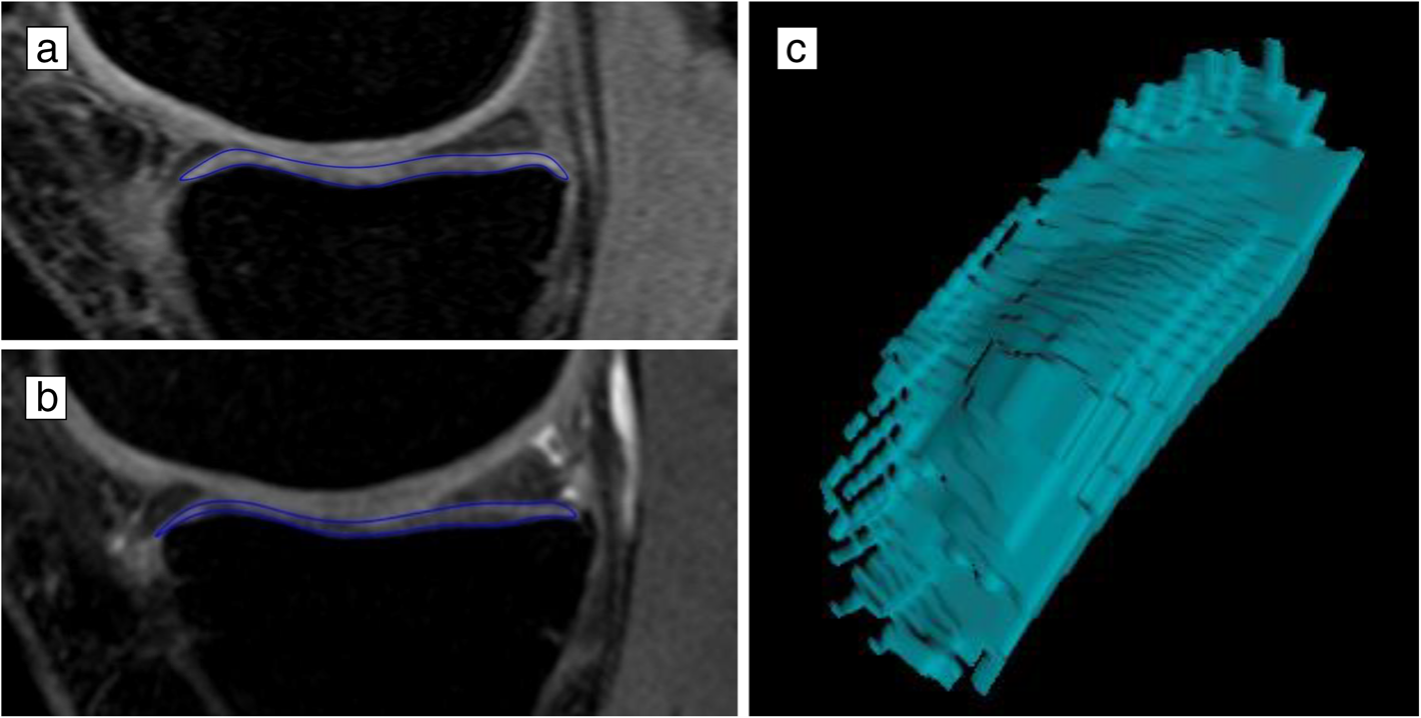
Examples of change in tibial cartilage volume at baseline (**a**) and follow-up (**b**) and 3D representation of tibial cartilage segmentation (**c**)

MRI of the right knee was acquired using a 1.5-T whole-body MRI unit (Picker, Cleveland, OH, USA) at baseline (2002–2004) and another 1.5-T whole-body MRI unit (Siemens, Espree, Pennsylvania, USA) at 10.7 years (2013–2015) due to the decommissioning of the old MRI in 2007. Both MRI units used a standard commercial transmit-receive extremity coil. MRI assessments for each participant were done according to a prespecified protocol. Tibial (medial and lateral) cartilage volume at baseline and 10.7 years follow-up was assessed using T1-weighted fat-suppressed three-dimensional gradient recall acquisition in the steady state, flip angle 55°, repetition time 58 msec, echo time 12 msec, field of view 16 cm, 60 partitions and 512 × 512 matrix. Sagittal images were obtained at a partition thickness of 1.5 mm and an in-plane resolution of 0.31 × 0.31 mm. The measurements were conducted in a paired fashion with known chronology by a trained reader using OsiriX software for Mac (University of Geneva, Geneva). The volumes of individual cartilage plates (medial tibia and lateral tibia) were isolated from the total volume by manually drawing disarticulation contours around the cartilage boundaries on a section by section basis. These data were then resampled by means of bilinear and cubic interpolation (area of 0.31 × 0.31 mm and 1.5 mm thickness, continuous sections) for the final 3D rendering (Fig. 1). The intra-observer coefficient of variation (CV) for cartilage volume measures was 2.1% for the medial tibial and 2.2% for the lateral tibial [15].

Tibial plateau bone area at the medial and tibial compartments was also measured on T1-weighted MRI and defined as the cross-sectional surface area of the tibial plateau. The CV for intra-observer repeatability was 2.2–2.6% [36].

### Radiographic OA

A standing anteroposterior semi-flexed view of the right knee with 15° of fixed knee flexion was performed for all participants at baseline. Joint space narrowing (JSN) and osteophytes were graded on a scale of 0–3 according to the Osteoarthritis Research Society International (OARSI) atlas [37], and radiographic OA was defined as the presence of any JSN or osteophytes. Severe radiographic OA in this study was defined as having either grade 3 JSN or grade 3 osteophytes at any of the medial and lateral sites.

### Clinical symptoms

At baseline and each follow-up, knee pain was assessed using the Western Ontario and McMaster Universities Osteoarthritis Index (WOMAC) pain subscale with total scores ranging from 0 (no pain) to 45 (worst pain) [38]. Functional disability was assessed using the WOMAC function subscale with total scores ranging from 0 (no disability) to 153 (worst disability). Each item of the WOMAC pain and function subscale was assessed using a 0–9 numeric rating scale, where 0 indicates no pain or function disability and 9 severe pain or function disability. The presence of knee pain and functional disability was defined as WOMAC pain and function scores larger than 0, respectively.

### Anthropometrics

Height and weight were measured at the baseline clinic visit, and BMI was calculated by weight divided by height squared (kg/m^2^). Weight change over 10.7 years was classified as weight loss, weight gain and stable weight with a commonly used cut point of 5 kg [39].

### Physical activity

Physical activity was evaluated as steps/day using pedometer (Omron HJ–003 and HJ-102, Omron Healthcare, Kyoto, Japan) at baseline, 2.7- and 5-year follow-up. Each participant was instructed to wear a pedometer for seven consecutive days. This was repeated 6 months later to account for seasonal variation. Mean steps/day was calculated as the average of the days worn at both time points [40].

### Other measures

Participants were asked if they had ever been diagnosed by a doctor as having rheumatoid arthritis at baseline. History of knee surgery (other than knee replacement) was recorded at baseline while history of knee injury was evaluated at the 2.7-year follow-up. Moreover, new incidents of knee surgery were assessed during the course of the study by asking “since your last interview, have you had any knee surgery?”. New knee injury was not recorded.

### Statistical analysis

Baseline characteristics were shown as percentage, mean (standard deviation [SD]) or median (interquartile range [IQR]) as appropriate. The characteristics of age groups (group 1, 50–60 years; group 2, 60–70 years; and group 3, 70–80 years) were compared using chi-square tests, analysis of variance or the Kruskal-Wallis test. We calculated both absolute (mm^3^/year) and percentage loss (%/year) of cartilage volume per annum, which were calculated as (a) absolute loss per annum = (baseline cartilage volume − follow-up cartilage volume)/time between two scans in years, and (b) percentage loss per annum = 100 × [(baseline cartilage volume − follow-up cartilage volume)/baseline cartilage volume]/time between two scans in years.

Linear regression analysis was performed to assess the association of age and BMI with loss of cartilage volume from baseline to 10.7 years with adjustment for potential confounders at baseline (sex, BMI, radiographic OA, history of knee surgery/injury, physical activity and site-specific tibial cartilage volume). The estimated coefficients with 95% confidence intervals were presented for loss of cartilage volume per 1 year older in age or one unit higher in BMI. In addition, non-linear associations of age and BMI with tibial cartilage loss were assessed by using locally weighted regression smoothing. Cartilage loss among different age groups (i.e. group 1, 50–60 years; group 2, 60–70 years; and group 3, 70–80 years) and groups of weight change (i.e. group 1, weight loss > 5 kg; group 2, weight change < 5 kg; group 3, weight gain > 5 kg) was also compared in the regression models. Because of the potential sex differences for the association between age and cartilage volume loss from previous evidence [1], stratified analysis by sex was prespecified. Despite this, we checked the interaction between age and sex for the annual loss of cartilage volume by adding an age × sex term in regression models. Moreover, the interactions between each of age and BMI with the presence and absence of radiographic OA (i.e. age × radiographic OA and BMI × radiographic OA, respectively) and between BMI and sex (i.e. BMI × sex) were assessed, but no significant interactions were found.

Four sensitivity analyses were conducted in this study. First, to address the missing data (0.2 to 8.1% missing) of covariates, multiple imputations were carried out. Twenty imputations were performed using baseline variables with complete data assuming missing at random. Baseline characteristics of participants included in and excluded from the study were compared. Second, participants were excluded if they had either severe radiographic OA or rheumatoid arthritis at baseline, considering the potential impact of severe radiographic OA and comorbidity of rheumatoid arthritis on the progression of cartilage loss. Third, the association between age and tibial cartilage loss was conducted by further adjusting for the site-specific tibial bone size and change in BMI, given that evidence has shown a potential effect of them on cartilage volume loss [41, 42]. Moreover, having new surgeries in the knee (not knee replacement) during the course of the study was also adjusted for as the fourth sensitivity analysis.

All statistical analyses were performed using Stata (version 15.1, StataCorp, TX, USA). Statistical significance was set at a *p* value of ≤ 0.05 (two-tailed).

## Results

### Participants

A total of 481 participants (49% female, mean age 60.8 ± 6.3 years [range 51.1–79.7]) had paired MRI imaging at baseline and 10.7 years. Baseline characteristics between participants were split by 3 age groups (Table 1). There were no significant differences in sex, BMI, knee pain and function scores; tibial bone size; and the prevalence of radiographic OA (including JSN and osteophytes) among groups. Tibial cartilage volume (mm^3^) at baseline was smaller in the oldest age group (aged 70–80 years), and physical activity (steps/day) was reduced with increasing age.

**Table 1:** Characteristics of study population†

	Age 50–60 years (*n* = 256)	Age 60–70 years (*n* = 181)	Age 70–80 years (*n* = 44)	*p* value^‡^
Age (year)	55.9 (2.3)	64.7 (3.0)	73.3 (2.3)	*< 0.001*
Females, %	52	48	45	0.604
BMI (kg/m^2^)	27.9 (4.8)	27.4 (3.9)	27.6 (3.9)	0.580
Radiographic OA, %	56	59	72	0.170
Joint space narrowing	56	58	72	0.173
Osteophytes	6	11	5	0.128
Tibial bone size (mm^2^)	3279.9 (469.5)	3366.6 (498.0)	3382.1 (558.5)	0.190
WOMAC pain score (0–45), median (IQR)	0.5 (0 to 4)	0 (0 to 3)	1 (0 to 4)	0.541
Any pain, %	50	46	52	0.612
WOMAC function score (0–153), median (IQR)	0 (0 to 8.5)	1 (0 to 7)	4.5 (0 to 16)	0.095
Any functional disability, %	49	53	68	0.057
History of knee surgery, %	11	9	7	0.741
History of knee injury, %*	14	10	9	0.413
Physical activity (steps/day)	9639.8 (3239.9)	9172.3 (3128.9)	7625.0 (2803.0)	*< 0.001*
Cartilage volume (mm^3^)				
Medial tibial	1509.5 (398.1)	1523.8 (449.3)	1438.6 (397.0)	0.479
Lateral tibial	2096.4 (591.5)	2046.9 (651.5)	1803.2 (599.9)	*0.015*
Total tibial	3606.0 (917.1)	3570.7 (1018.6)	3241.8 (907.5)	0.065

*BMI* body mass index, *IQR* interquartile range, *OA* osteoarthritis, *WOMAC* Western Ontario and McMaster Universities Osteoarthritis Index

^†^Data are presented as mean (standard deviation) unless specified otherwise (e.g. percentage, median (IQR)). Italicised data denotes statistically significant result

^‡^*p* values are calculated using chi-square tests, analysis of variance or the Kruskal-Wallis test

*History of knee injury was assessed at the 2.7 -year follow-up

### Age and tibial cartilage volume loss

**Fig. 2:**
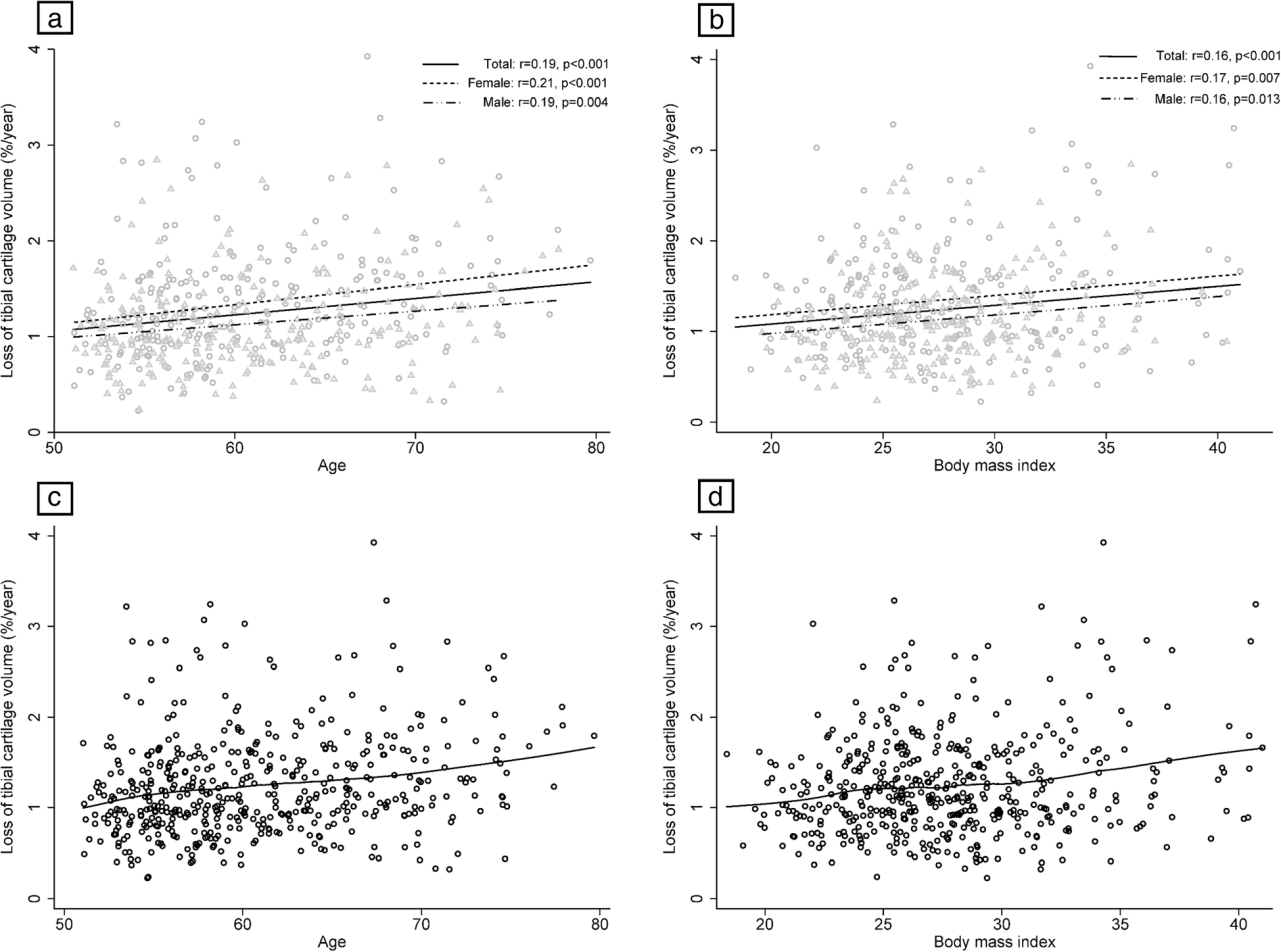
Linear (**a**, **b**; triangle indicates males and circle females) and non-linear (**c**, **d**) associations of age and body mass index at baseline with loss of tibial cartilage volume (%/year)

The average rate of loss of total tibial cartilage volume was 1.2% per annum (range 0.2 to 3.9%) with 100% of participants losing tibial cartilage volume over the study period. There was a positive correlation between age and loss of tibial cartilage volume, with older adults losing more tibial cartilage volume per year (%/year, Fig. 2a); similar results were found for the correlation between BMI at baseline and loss of tibial cartilage volume (%/year, Fig. 2b). No evidence of a non-linear association between age or BMI and tibial cartilage loss (%/year) was observed (Fig. 2c, d). Additional file 1: Figure S1 shows the association of age and BMI with absolute loss of tibial cartilage volume per year (mm^3^/year). In multivariable analysis (Table 2), age was significantly associated with loss of tibial cartilage volume at both the medial and lateral compartments, independent of radiographic OA. There was a significant interaction between age and sex for lateral tibial cartilage volume loss. In stratified analysis, females lost more cartilage from all compartments with increasing age; however, the association for males was only significant for medial and total tibial cartilage loss.

**Table 2:** Association of age and sex with tibial cartilage volume change over 10.7 years

	Multivariable, β (95% CI)			Interaction with sex (male vs. female)	*p* for interaction
	Females (*n* = 211)^†^	Males (*n* = 217)^†^	Combined (*n* = 428)^‡^		
Loss of cartilage volume (mm^3^/year)					
Medial tibial	*0.27 (0.00 to 0.54)*	*0.43 (0.14 to 0.73)*	*0.36 (0.16 to 0.57)*	0.16 (− 0.24 to 0.57)	0.430
Lateral tibial	*0.37 (0.11 to 0.63)*	0.04 (− 0.27 to 0.34)	0.18 (− 0.02 to 0.38)	− 0.33 (− 0.73 to 0.07)	0.107
Total tibial	*0.66 (0.24 to 1.07)*	*0.49 (0.05 to 0.92)*	*0.56 (0.26 to 0.86)*	− 0.17 (− 0.78 to 0.43)	0.575
Loss of cartilage volume (%/year)					
Medial tibial	*0.023 (0.003 to 0.043)*	*0.023 (0.006 to 0.041)*	*0.023 (0.010 to 0.036)*	0.000 (− 0.026 to 0.027)	0.980
Lateral tibial	*0.025 (0.009 to 0.041)*	0.002 (− 0.010 to 0.015)	*0.013 (0.003 to 0.023)*	*− 0.023 (− 0.043 to − 0.003)*	*0.024*
Total tibial	*0.024 (0.010 to 0.038)*	*0.012 (0.002 to 0.022)*	*0.018 (0.009 to 0.026)*	− 0.012 (− 0.029 to 0.005)	0.173

^†^Model 1: adjusted for body mass index, radiographic osteoarthritis, history of knee surgery and knee injury, physical activity and site-specific tibial cartilage volume at baseline

^‡^Model 1 + sex

Italicised data denotes statistically significant result

**Fig. 3:**
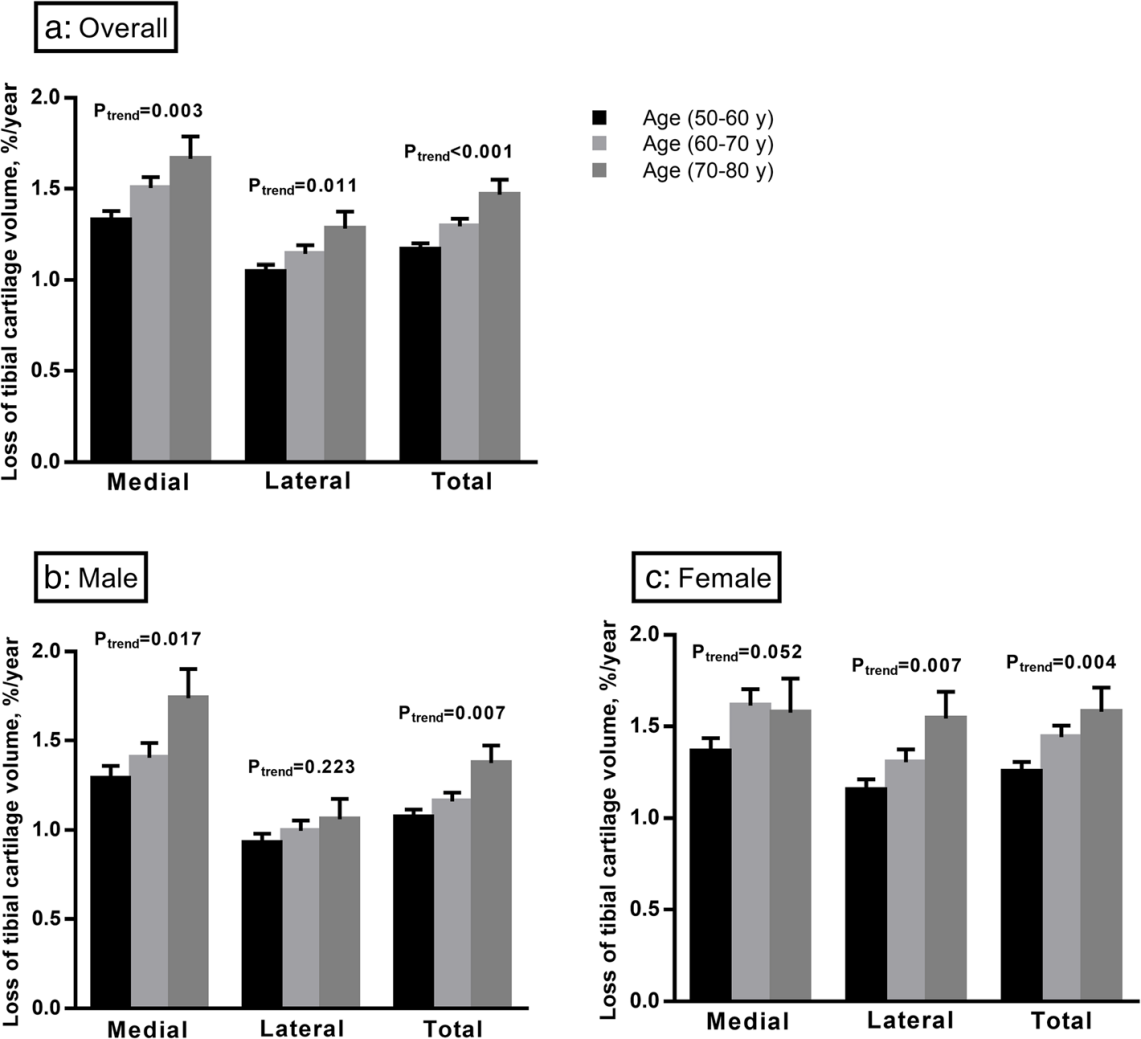
Loss of tibial cartilage volume among age groups over 10.7 years overall (**a**) and in males (**b**) and females (**c**). Bar graph indicates the mean value of tibial cartilage loss (%/year), and error bars indicate standard errors. *p* for trend was calculated by univariable linear regression models

Comparing participants by age groups (Fig. 3 and Additional file 1: Figure S2), there was a significant trend that older participants lost more tibial cartilage volume at both medial and lateral compartments. These results persisted after further adjustment for potential confounders (Table 3). Of note, there was a trend to a significant interaction between age group and sex for loss of lateral tibial cartilage (*p* = 0.084), and females lost more lateral tibial cartilage volume with increasing age compared to males. No significant interaction between age group and sex was observed for cartilage loss at the medial or total tibia. In prespecified stratified analysis, females lost more medial and lateral cartilage volume with increasing age group, but in males, there was a significantly greater medial and total tibial cartilage loss with increasing age group and no association for cartilage loss at the lateral compartment.

**Table 3:** Association of age group and sex with loss of tibial cartilage volume (%/year) over 10.7 years

	Multivariable, β (95% CI)		
	Medial	Lateral	Total tibia
Combined (*n* = 428)^†^			
Age 50–60 years	Ref.	Ref.	Ref.
Age 60–70 years	0.16 (− 0.01 to 0.33)	0.07 (− 0.05 to 0.20)	*0.11 (0.00 to 0.22)*
Age 70–80 years	*0.38 (0.08 to 0.67)*	*0.31 (0.08 to 0.53)*	*0.35 (0.16 to 0.54)*
*p* for trend	*0.006*	*0.012*	*< 0.001*
Females (*n* = 211)^‡^			
Age 50–60 years	Ref.	Ref.	Ref.
Age 60–70 years	*0.26 (0.01 to 0.51)*	0.11 (− 0.09 to 0.31)	0.17 (− 0.01 to 0.34)
Age 70–80 years	0.29 (− 0.19 to 0.76)	*0.60 (0.22 to 0.98)*	*0.47 (0.13 to 0.80)*
*p* for trend	*0.042*	*0.007*	*0.004*
Males (*n* = 217)^‡^			
Age 50–60 years	Ref.	Ref.	Ref.
Age 60–70 years	0.09 (− 0.14 to 0.32)	0.02 (− 0.14 to 0.18)	0.05 (− 0.08 to 0.19)
Age 70–80 years	*0.41 (0.03 to 0.79)*	0.12 (− 0.15 to 0.39)	*0.27 (0.05 to 0.49)*
*p* for trend	0.053	0.449	*0.035*
Interaction with sex	− 0.04 (− 0.29 to 0.21)	− 0.17 (− 0.36 to 0.02)	− 0.10 (− 0.26 to 0.07)
*p* for interaction	0.778	0.084	0.241

^†^Model 1: adjusted for sex, BMI, radiographic osteoarthritis, history of knee surgery and knee injury, physical activity and site-specific tibial cartilage volume at baseline

^‡^Model 2: adjusted for BMI, radiographic osteoarthritis, history of knee surgery and knee injury, physical activity and site-specific tibial cartilage volume at baseline

Italicised data denotes statistically significant results

### Body mass index and tibial cartilage volume loss

**Fig. 4:**
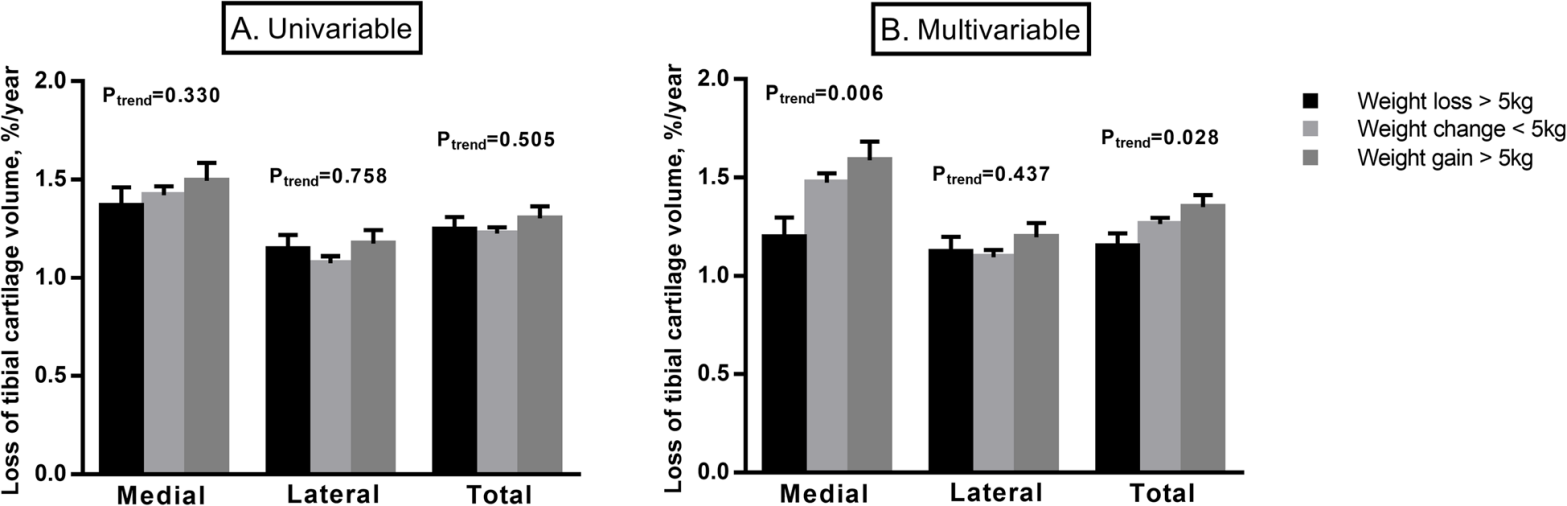
Uni- (**a**) and multivariable (**b**) analyses for the association between weight change and loss of tibial cartilage volume. Bar graph indicates the mean value of tibial cartilage loss (%/year), and error bars indicate standard errors. Multivariable analyses adjusted for age, sex, body mass index, radiographic osteoarthritis, history of knee surgery and knee injury, physical activity and site-specific tibial cartilage volume at baseline

There was a significant association between BMI at baseline and loss of cartilage volume at the medial but not lateral compartment. After adding change in BMI over time to the model, both BMI at baseline and change in BMI were associated with a greater loss of medial tibial cartilage volume (Table 4). Moreover, there was a significant association between weight change (by categories) and medial tibial cartilage loss in multivariable analysis (Fig. 4 and Additional file 1: Figure S3).

**Table 4:** Association of body mass index and change in body mass index with loss of tibial cartilage volume over 10.7 years (*n* = 428)

	Multivariable, β (95% CI)	Multivariable, β (95% CI)	
	BMI at baseline^†^	BMI at baseline^‡^	Change in BMI^‡^
Loss of cartilage volume (mm^3^/year)			
Medial tibial	*0.55 (0.27 to 0.84)*	*0.58 (0.29 to 0.86)*	*0.85 (0.26 to 1.43)*
Lateral tibial	0.12 (− 0.15 to 0.40)	0.14 (− 0.14 to 0.42)	0.38 (− 0.20 to 0.95)
Total tibial	*0.69 (0.27 to 1.12)*	*0.73 (0.31 to 1.15)*	*1.20 (0.33 to 2.07)*
Loss of cartilage volume (%/year)			
Medial tibial	*0.038 (0.020 to 0.056)*	*0.040 (0.022 to 0.058)*	*0.055 (0.018 to 0.093)*
Lateral tibial	0.008 (− 0.005 to 0.022)	0.009 (− 0.005 to 0.023)	0.022 (− 0.007 to 0.050)
Total tibial	*0.022 (0.011 to 0.034)*	*0.023 (0.012 to 0.035)*	*0.035 (0.010 to 0.059)*

^†^Model 1: adjusted for age, sex, radiographic osteoarthritis, history of knee surgery and knee injury, physical activity and site-specific tibial cartilage volume at baseline

^‡^Model 1 + change in BMI over 10.7 years

Italicised data denotes statistically significant results. *BMI* body mass index, *CI* confidence interval

### Sensitivity analysis

In sensitivity analysis, multiple imputations for missing data produced similar results, except that the association between age group and loss of medial and total tibial cartilage volume became statistically significant in males (Additional file 1: Table S1-S3). Baseline characteristics of participants included in the study were similar to those who were not, although participants included were younger and had less knee pain and functional disability (Additional file 1: Table S4). The association between age or age group and loss of tibial cartilage volume was attenuated in females, after excluding participants who had rheumatoid arthritis (*n* = 39, 8.1%) and/or severe radiographic OA (*n* = 10, 2.1%) at baseline (Additional file 1: Table S5-S7). In multivariable analysis, further adjustment of site-specific bone size at baseline and change in BMI or new surgeries in the knee during the follow-up did not change the association between age or BMI and cartilage loss (data not shown).

## Discussion

In this 10.7-year longitudinal study of a population-based cohort, all participants lost tibial knee cartilage over time and increasing age was associated with a greater loss of tibial cartilage volume. In addition, the annual loss of cartilage volume was faster in females, particularly in the lateral compartment. Moreover, both BMI at baseline and increase in BMI over time were associated with a greater loss of tibial cartilage volume at the medial but not lateral compartment. The findings of this study reveal that tibial cartilage loss is universal in older adults and will become faster over time, particularly in those with higher BMI at baseline and increased BMI over time.

The results of this study contrast to reports based on radiographic findings where less than 50% of the older population have progressive joint space narrowing over 4 to 14 years [2–7]. This most likely reflects the higher sensitivity of MRI to change. Moreover, we found that cartilage loss appeared to increase with increasing age. This finding is consistent with two previous radiographic studies conducted in a similar [2] or younger [3] population but contrasts to others [4, 7] that suggested a similar rate of change in radiographs among age groups. The inconsistency is most likely due to that radiographic joint space is only a surrogate measure of articular cartilage thickness, such that a small change in knee cartilage volume may not be captured in radiographs. Furthermore, radiographic joint space width can be substantially influenced by the position of the knee and meniscal pathology [43], leading to misclassification.

In this population-based older adult cohort, we observed that over a 10.7-year period, the older the subject, the greater the cartilage volume loss implying that annual cartilage loss is not constant but increases over time. Similarly, in another older cohort of OA patients, older patients at baseline experienced a greater loss of tibial cartilage volume over 2 years [19]. Moreover, this association of age with an increased rate of cartilage volume loss has been demonstrated over a 2-year period in a younger population-based cohort with a maximum age of 60 (mean age 45 years vs. 60.8 years in the current study) [1]. The rate of tibial cartilage volume loss was lower in our cohort at − 1.2% per annum compared to the younger cohort (− 3.0% per annum), but half of the younger cohort was selected based on family risk of osteoarthritis, and these offspring of OA patients have a higher rate of cartilage loss [44] which most likely contributes to the higher rates demonstrated in the younger cohort. In addition, the longer follow-up of our cohort may provide a more reliable representation of the real rate of cartilage loss over time. Indeed, several longitudinal studies with a short- or medium-term follow-up have observed an increased cartilage thickness at the medial site of tibiofemoral joint and hypothesised that cartilage swelling and softening prior to MRI-detected cartilage loss may play a role [28, 29]. Moreover, one study found cartilage thickening at the medial femur and cartilage thinning at trochlea of the femur [29], indicating a variation of cartilage change among anatomic sites.

Females appeared to lose cartilage at a faster rate than men, especially in the lateral compartment. With adjustment for other factors including cartilage volume at baseline, females lost cartilage at a faster rate with age in both the medial and lateral compartments, whereas males did not appear to lose much cartilage from the lateral tibial compartment. The sex difference in lateral tibial cartilage volume loss may be related to the anatomical variations between knees in men and women. Women have been shown to have a greater valgus knee angle [45] compared to men, which is associated with an increased risk of lateral compartment cartilage loss [46, 47]. A higher prevalence of OA [22, 48], cartilage defects [49] and rate of cartilage loss [1] in women has been reported in other studies. It may reflect a lower baseline cartilage volume in females as well as an increased rate of cartilage loss in postmenopausal females [15]. When divided by age groups, the age association for females was statistically significant for both medial and lateral compartments, which may reflect this postmenopausal increase in the rate of cartilage loss. Moreover, it is important to note that after excluding participants with either rheumatoid arthritis or severe radiographic OA at baseline, the association between age and tibial cartilage loss in females was attenuated (excluding severe radiographic OA only did not lead to these changes). This may suggest that the increased tibial cartilage loss with ageing in females was partly driven by the effect of rheumatoid arthritis on bone and cartilage damage [50]. However, this needs to be confirmed in future studies given that data on rheumatoid arthritis were self-reported and the association between age and cartilage loss was not changed in males.

This study indicated that higher BMI at baseline was a risk factor of structural OA progression over 10.7 years, showing a linear relationship with tibial cartilage volume loss in both males and females. Moreover, we found an association between change in BMI and loss of tibial cartilage volume at the medial but not tibial compartment, independent of age and BMI at baseline. In addition, there was a significant association between weight change and medial tibial cartilage volume loss. This agrees with previous studies demonstrating that weight change was associated with loss of tibial cartilage volume at the medial but not lateral compartment in overweight and obese adults over 2.3 to 8 years [32–34, 41, 51]. It remains unclear why a significant association between change in BMI or weight and tibial cartilage loss was only observed at the medial compartment. One potential explanation is that the medial compartment undertakes greater weight-bearing loads and has a higher rate of cartilage loss (as observed in this study); therefore, any effect of weight change on cartilage would be greater at the medial compartment [51]. Moreover, varus knee would also increase the loading to the medial compartment, but knee alignment was not measured in this cohort and this cannot be assessed.

The main strength of our study is the long follow-up of a community-based cohort which is likely to be representative of the general population. To our knowledge, this is the longest follow-up cohort looking at the rate of change in knee cartilage volume as measured by MRI. There are several limitations in this study. First, we did not measure femoral cartilage volume. However, Cicuttini et al. [52] found a strong correlation between longitudinal changes in tibial and femoral cartilage volume (*r* = 0.81 and 0.71 in the medial and lateral sites, respectively) in a similar population (mean age 63.7 years, 58% female) and they suggested that measuring tibial cartilage alone may be adequate to evaluate structural change in the tibiofemoral joint. Second, the known chronology for the measurements of tibial cartilage volume may have introduced an observer-expectancy bias since loss of tibial cartilage volume was found in all participants. Nonetheless, it has been shown that scoring without known chronology substantially decreases sensitivity in detecting clinically relevant changes [53, 54]. In addition, the known chronology for MRI readings is unlikely to bias the association of age, sex and BMI with tibial cartilage loss given that demographic information of study participants was not available to the MRI reader. Third, cartilage volume was measured in only 43.8% (481/1099) of participants in the TASOAC study for the 10.7-year follow up, suggesting a potential attrition bias. While baseline characteristics such as sex, BMI and radiographic OA were similar between included participants and those who were lost to follow-up, included participants were younger (60.8 vs. 64.7 years), were physically more active and had milder knee symptoms at baseline and a lower rate of history of knee surgery, indicating that the study may represent a slightly younger and healthier population. Lastly, tibial cartilage volume was measured at only two time points in this study, and this prevented us from investigating the course of tibial cartilage volume. While our study suggested a linear increase in tibial cartilage loss over time (ageing), this finding needs to be confirmed in studies with MRI measures at multiple time points.

## Conclusion

Knee cartilage volume declines at a faster rate with increasing age and BMI in both males and females, particularly in the medial compartment. In contrast to the slow rate of change in radiographs, our findings suggest that cartilage loss at the knee is universal in this age group.

## Supplementary information

**Additional file 1 : Table S1.** Association of age and sex with loss of tibial cartilage volume over 10.7 years after multiple imputations for missing data. **Table S2.** Association between age group and loss of tibial cartilage volume (%/year) over 10.7 years after multiple imputations for missing data. **Table S3.** Association of body mass index and change in body mass index with loss of tibial cartilage volume over 10.7 years after multiple imputations for missing data. **Table S4.** Characteristics of participants included in and excluded from the study. **Table S5.** Association of age and sex with loss of tibial cartilage volume over 10.7 years after excluding participants with rheumatoid arthritis or severe radiographic osteoarthritis. **Table S6.** Association between age group and loss of tibial cartilage volume (%/year) over 10.7 years after excluding participants with rheumatoid arthritis or severe radiographic osteoarthritis. **Table S7.** Association of body mass index and change in body mass index with loss of tibial cartilage volume over 10.7 years after excluding participants with rheumatoid arthritis or severe radiographic osteoarthritis. **Figure S1.** Linear (A and B, triangle indicates males and circle females) and non-linear (C and D) associations of age and body mass index at baseline with loss of tibial cartilage volume (mm^3^/year). **Figure S2.** Loss of tibial cartilage volume among age groups over 10.7 years overall (A) and in males (B) and females (C). **Figure S3.** Uni- (A) and multivariable (B) analyses for the association between weight change and loss of tibial cartilage volume.

## Data Availability

All data generated or analysed during this study are included in this published article.

## Abbreviations

BMI: Body mass index
CV: Coefficient of variation
IQR: Interquartile range
JSN: Joint space narrowing
MRI: Magnetic resonance imaging
OA: Osteoarthritis
OARSI: The Osteoarthritis Research Society International
SD: Standard deviation
TASOAC: The Tasmania Older Adult Cohort
WOMAC: The Western Ontario and McMaster Universities Osteoarthritis Index
