# Supplementary material for: Association of age, sex and BMI with the rate of change in tibial cartilage volume: a 10.7-year longitudinal cohort study
Source: Arthritis Res Ther. 2019 Dec 9;21:273. doi: 10.1186/s13075-019-2063-z (PMC6902563; doi:10.1186/s13075-019-2063-z)
Supplement: Supplementary file 1 — Additional file 1 : Table S1. Association of age and sex with loss of tibial cartilage volume over 10.7 years after multiple imputations for missing data. Table S2. Association between age group and loss of tibial cartilage volume (%/year) over 10.7 years after multiple imputations for missing data. Table S3. Association of body mass index and change in body mass index with loss of tibial cartilage volume over 10.7 years after multiple imputations for missing data. Table S4. Characteristics of participants included in and excluded from the study. Table S5. Association of age and sex with loss of tibial cartilage volume over 10.7 years after excluding participants with rheumatoid arthritis or severe radiographic osteoarthritis. Table S6. Association between age group and loss of tibial cartilage volume (%/year) over 10.7 years after excluding participants with rheumatoid arthritis or severe radiographic osteoarthritis. Table S7. Association of body mass index and change in body mass index with loss of tibial cartilage volume over 10.7 years after excluding participants with rheumatoid arthritis or severe radiographic osteoarthritis. Figure S1. Linear (A and B, triangle indicates males and circle females) and non-linear (C and D) associations of age and body mass index at baseline with loss of tibial cartilage volume (mm3/year). Figure S2. Loss of tibial cartilage volume among age groups over 10.7 years overall (A) and in males (B) and females (C). Figure S3. Uni- (A) and multivariable (B) analyses for the association between weight change and loss of tibial cartilage volume. [file 13075_2019_2063_MOESM1_ESM.docx]

Table S1. Association of age and sex with loss of tibial cartilage volume over 10.7 years after multiple imputations for missing data.

|  | Multivariable, β (95% CI) | | | Interaction with sex (male vs. female) | *P* for interaction |
| --- | --- | --- | --- | --- | --- |
|  | Females (n=238) † | Males (n=243) † | Combined (n=481) ‡ |  |  |
| Loss of cartilage volume (mm^3^/year) |  |  |  |  |  |
| Medial tibial | **0.25 (0.01 to 0.49)** | **0.43 (0.16 to 0.71)** | **0.34 (0.15 to 0.52)** | 0.19 (-0.18 to 0.55) | 0.319 |
| Lateral tibial | **0.29 (0.06 to 0.52)** | 0.06 (-0.22 to 0.34) | 0.16 (-0.02 to 0.35) | -0.22 (-0.59 to 0.14) | 0.225 |
| Total tibial | **0.54 (0.16 to 0.91)** | **0.51 (0.10 to 0.92)** | **0.51 (0.24 to 0.79)** | -0.02 (-0.58 to 0.53) | 0.933 |
| Loss of cartilage volume (%/year) |  |  |  |  |  |
| Medial tibial | **0.021 (0.003 to 0.039)** | **0.024 (0.008 to 0.040)** | **0.022 (0.010 to 0.034)** | 0.002 (-0.021 to 0.026) | 0.839 |
| Lateral tibial | **0.019 (0.005 to 0.033)** | 0.003 (-0.008 to 0.015) | **0.011 (0.002 to 0.020)** | -0.016 (-0.034 to 0.002) | 0.084 |
| Total tibial | **0.020 (0.007 to 0.032)** | **0.013 (0.003 to 0.022)** | **0.016 (0.008 to 0.024)** | -0.007 (-0.022 to 0.009) | 0.401 |

† Model 1: adjusted for body mass index, radiographic osteoarthritis, history of knee surgery and knee injury, physical activity and site-specific tibial cartilage volume at baseline.

‡ Model 2: Model 1 + sex.

Bold denotes statistically significant results. BMI, body mass index; CI, confidence interval.

Table S2. Association between age group and loss of tibial cartilage volume (%/year) over 10.7 years after multiple imputations for missing data.

|  | Multivariable, β (95% CI) | | |
| --- | --- | --- | --- |
|  | Medial | Lateral | Total tibia |
| **Combined (n=481)†** |  |  |  |
| Age 50-60 y | Ref. | Ref. | Ref. |
| Age 60-70 y | **0.19 (0.04 to 0.35)** | 0.10 (-0.01 to 0.22) | **0.14 (0.04 to 0.24)** |
| Age 70-80 y | **0.32 (0.05 to 0.58)** | **0.22 (0.01 to 0.42)** | **0.27 (0.10 to 0.45)** |
| P for trend | **0.003** | **0.015** | **0.001** |
| **Females (n=238)‡** |  |  |  |
| Age 50-60 y | Ref. | Ref. | Ref. |
| Age 60-70 y | **0.27 (0.04 to 0.50)** | 0.14 (-0.04 to 0.32) | **0.18 (0.02 to 0.35)** |
| Age 70-80 y | 0.24 (-0.16 to 0.64) | **0.35 (0.03 to 0.68)** | **0.30 (0.01 to 0.59)** |
| P for trend | **0.036** | **0.020** | **0.009** |
| **Males (n=243)‡** |  |  |  |
| Age 50-60 y | Ref. | Ref. | Ref. |
| Age 60-70 y | 0.14 (-0.07 to 0.35) | 0.06 (-0.09 to 0.21) | 0.09 (-0.03 to 0.22) |
| Age 70-80 y | **0.39 (0.04 to 0.74)** | 0.11 (-0.14 to 0.36) | **0.25 (0.04 to 0.46)** |
| P for trend | **0.025** | 0.315 | **0.015** |
| **Interaction with sex** | -0.009 (-0.24 to 0.22) | -0.11 (-0.28 to 0.06) | -0.05 (-0.20 to 0.10) |
| ***P* for interaction** | 0.936 | 0.221 | 0.503 |

† Model 1: adjusted for sex, body mass index, radiographic osteoarthritis, history of knee surgery and knee injury, physical activity and site-specific tibial cartilage volume at baseline.

‡ Model 2: adjusted for body mass index, radiographic osteoarthritis, history of knee surgery and knee injury, physical activity and site-specific tibial cartilage volume at baseline.

Bold denotes statistically significant results. CI, confidence interval.

Table S3. Association of body mass index and change in body mass index with loss of tibial cartilage volume over 10.7 years after multiple imputations for missing data.

|  | Multivariable, β (95% CI) |  | Multivariable, β (95% CI) | |
| --- | --- | --- | --- | --- |
|  | BMI at baseline † |  | BMI at baseline ‡ | Change in BMI ‡ |
| Loss of cartilage volume (mm^3^/year) |  |  |  |  |
| Medial tibial | **0.50 (0.24 to 0.76)** |  | **0.53 (0.27 to 0.79)** | **0.69 (0.15 to 1.22)** |
| Lateral tibial | 0.09 (-0.17 to 0.34) |  | 0.10 (-0.15 to 0.36) | 0.36 (-0.17 to 0.88) |
| Total tibial | **0.61 (0.22 to 1.00)** |  | **0.66 (0.27 to 1.05)** | **1.02 (0.22 to 1.83)** |
| Loss of cartilage volume (%/year) |  |  |  |  |
| Medial tibial | **0.036 (0.019 to 0.053)** |  | **0.038 (0.021 to 0.055)** | **0.041 (0.007 to 0.076)** |
| Lateral tibial | 0.005 (-0.008 to 0.018) |  | 0.006 (-0.007 to 0.019) | 0.020 (-0.006 to 0.046) |
| Total tibial | **0.019 (0.008 to 0.030)** |  | **0.021 (0.010 to 0.032)** | **0.028 (0.005 to 0.051)** |

† Model 1: adjusted for age, sex, radiographic osteoarthritis, history of knee surgery and knee injury, physical activity and site-specific tibial cartilage volume at baseline.

‡ Model 1 + Change in BMI over 10.7 years.

Bold denotes statistically significant result. BMI, body mass index; CI, confidence interval.

Table S4. Characteristics of participants included in and excluded from the study

|  | Included (n=481) | Excluded (n=618) | p value ‡ |
| --- | --- | --- | --- |
| Age (y) | 60.8 (6.3) | 64.7 (7.9) | **<0.001** |
| Females, % | 49 | 52 | 0.332 |
| BMI (kg/m^2^) | 27.7 (4.4) | 28.1 (5.0) | 0.179 |
| Radiographic OA, % (n=1018) | 58 | 61 | 0.408 |
| Joint space narrowing | 58 | 60 | 0.421 |
| Osteophytes | 8 | 11 | 0.055 |
| Tibial bone area (mm^2^) (n=814) | 3321.2 (489.3) | 3300.6 (498.1) | 0.552 |
| WOMAC pain score (0-45), median (IQR) | 0 (0 to 4) | 1 (0 to 6) | **<0.001** |
| Any pain, % | 49 | 57 | **<0.001** |
| WOMAC function score (0-153), median (IQR) | 1 (0 to 9) | 4 (0 to 18) | **<0.001** |
| Any functional disability, % | 52 | 63 | **<0.001** |
| History of knee surgery, % | 10 | 14 | **0.040** |
| History of knee injury, % * | 12 | 12 | 0.853 |
| Physical activity (steps/day) | 9285.0 (3205.9) | 8075.7 (3379.5) | **<0.001** |

BMI, body mass index; IQR, interquartile range; OA, osteoarthritis.

Data are presented as mean (standard deviation) unless specified otherwise (e.g. percentage).

Table S5. Association of age and sex with loss of tibial cartilage volume over 10.7 years after excluding participants with rheumatoid arthritis or severe radiographic osteoarthritis.

|  | Multivariable, β (95% CI) | | | Interaction with sex (male vs. female) | *P* for interaction |
| --- | --- | --- | --- | --- | --- |
|  | Females (n=185) † | Males (n=199) † | Combined (n=384) ‡ |  |  |
| Loss of cartilage volume (mm^3^/year) |  |  |  |  |  |
| Medial tibial | 0.20 (-0.08 to 0.49) | **0.48 (0.17 to 0.78)** | **0.36 (0.15 to 0.57)** | 0.28 (-0.15 to 0.70) | 0.205 |
| Lateral tibial | 0.26 (-0.01 to 0.54) | 0.04 (-0.28 to 0.35) | 0.12 (-0.09 to 0.33) | -0.22 (-0.65 to 0.21) | 0.306 |
| Total tibial | **0.45 (0.01 to 0.89)** | **0.54 (0.08 to 1.00)** | **0.50 (0.18 to 0.81)** | 0.09 (-0.56 to 0.73) | 0.795 |
| Loss of cartilage volume (%/year) |  |  |  |  |  |
| Medial tibial | 0.018 (-0.004 to 0.040) | **0.026 (0.008 to 0.044)** | **0.023 (0.009 to 0.037)** | 0.008 (-0.020 to 0.036) | 0.587 |
| Lateral tibial | **0.020 (0.003 to 0.036)** | 0.002 (-0.011 to 0.015) | 0.009 (-0.001 to 0.019) | -0.018 (-0.038 to 0.003) | 0.097 |
| Total tibial | **0.018 (0.003 to 0.033)** | **0.013 (0.003 to 0.024)** | **0.015 (0.007 to 0.024)** | -0.004 (-0.022 to 0.014) | 0.643 |

† Model 1: adjusted for body mass index, radiographic osteoarthritis, history of knee surgery and knee injury, physical activity and site-specific tibial cartilage volume at baseline.

‡ Model 2: Model 1 + sex.

Bold denotes statistically significant results. BMI, body mass index; CI, confidence interval.

Table S6. Association between age group and loss of tibial cartilage volume (%/year) over 10.7 years after excluding participants with rheumatoid arthritis or severe radiographic osteoarthritis.

|  | Multivariable, β (95% CI) | | |
| --- | --- | --- | --- |
|  | Medial | Lateral | Total tibia |
| **Combined (n=384)†** |  |  |  |
| Age 50-60 y | Ref. | Ref. | Ref. |
| Age 60-70 y | 0.16 (-0.02 to 0.34) | 0.05 (-0.08 to 0.18) | 0.09 (-0.02 to 0.21) |
| Age 70-80 y | **0.39 (0.08 to 0.71)** | 0.22 (-0.02 to 0.45) | **0.31 (0.10 to 0.51)** |
| P for trend | **0.007** | 0.103 | **0.003** |
| **Females (n=185)‡** |  |  |  |
| Age 50-60 y | Ref. | Ref. | Ref. |
| Age 60-70 y | 0.17 (-0.10 to 0.44) | 0.08 (-0.12 to 0.29) | 0.10 (-0.08 to 0.29) |
| Age 70-80 y | 0.29 (-0.25 to 0.84) | **0.43 (0.01 to 0.85)** | 0.36 (-0.02 to 0.73) |
| P for trend | 0.145 | 0.076 | 0.061 |
| **Males (n=199)‡** |  |  |  |
| Age 50-60 y | Ref. | Ref. | Ref. |
| Age 60-70 y | 0.16 (-0.08 to 0.40) | 0.01 (-0.16 to 0.18) | 0.08 (-0.06 to 0.23) |
| Age 70-80 y | **0.43 (0.04 to 0.82)** | 0.12 (-0.16 to 0.39) | **0.28 (0.05 to 0.51)** |
| P for trend | **0.024** | 0.509 | **0.021** |
| **Interaction with sex** | 0.04 (-0.23 to 0.31) | -0.11 (-0.31 to 0.09) | -0.02 (-0.20 to 0.15) |
| ***P* for interaction** | 0.786 | 0.290 | 0.803 |

† Model 1: adjusted for sex, body mass index, radiographic osteoarthritis, history of knee surgery and knee injury, physical activity and site-specific tibial cartilage volume at baseline.

‡ Model 2: adjusted for body mass index, radiographic osteoarthritis, history of knee surgery and knee injury, physical activity and site-specific tibial cartilage volume at baseline.

Bold denotes statistically significant results. CI, confidence interval.

Table S7. Association of body mass index and change in body mass index with loss of tibial cartilage volume over 10.7 years after excluding participants with rheumatoid arthritis or severe radiographic osteoarthritis.

|  | Multivariable, β (95% CI) |  | Multivariable, β (95% CI) | |
| --- | --- | --- | --- | --- |
|  | BMI at baseline † |  | BMI at baseline ‡ | Change in BMI ‡ |
| Loss of cartilage volume (mm^3^/year) |  |  |  |  |
| Medial tibial | **0.51 (0.21 to 0.80)** |  | **0.53 (0.24 to 0.82)** | **0.94 (0.34 to 1.54)** |
| Lateral tibial | 0.20 (-0.09 to 0.50) |  | 0.22 (-0.08 to 0.51) | 0.41 (-0.20 to 1.01) |
| Total tibial | **0.72 (0.27 to 1.16)** |  | **0.76 (0.31 to 1.20)** | **1.34 (0.44 to 2.24)** |
| Loss of cartilage volume (%/year) |  |  |  |  |
| Medial tibial | **0.036 (0.017 to 0.056)** |  | **0.038 (0.019 to 0.057)** | **0.061 (0.022 to 0.099)** |
| Lateral tibial | 0.013 (-0.001 to 0.027) |  | 0.014 (0.000 to 0.028) | 0.025 (-0.004 to 0.055) |
| Total tibial | **0.024 (0.011 to 0.036)** |  | **0.025 (0.013 to 0.037)** | **0.039 (0.015 to 0.064)** |

† Model 1: adjusted for age, sex, radiographic osteoarthritis, history of knee surgery and knee injury, physical activity and site-specific tibial cartilage volume at baseline.

‡ Model 1 + Change in BMI over 10.7 years.

Bold denotes statistically significant result. BMI, body mass index; CI, confidence interval.

**
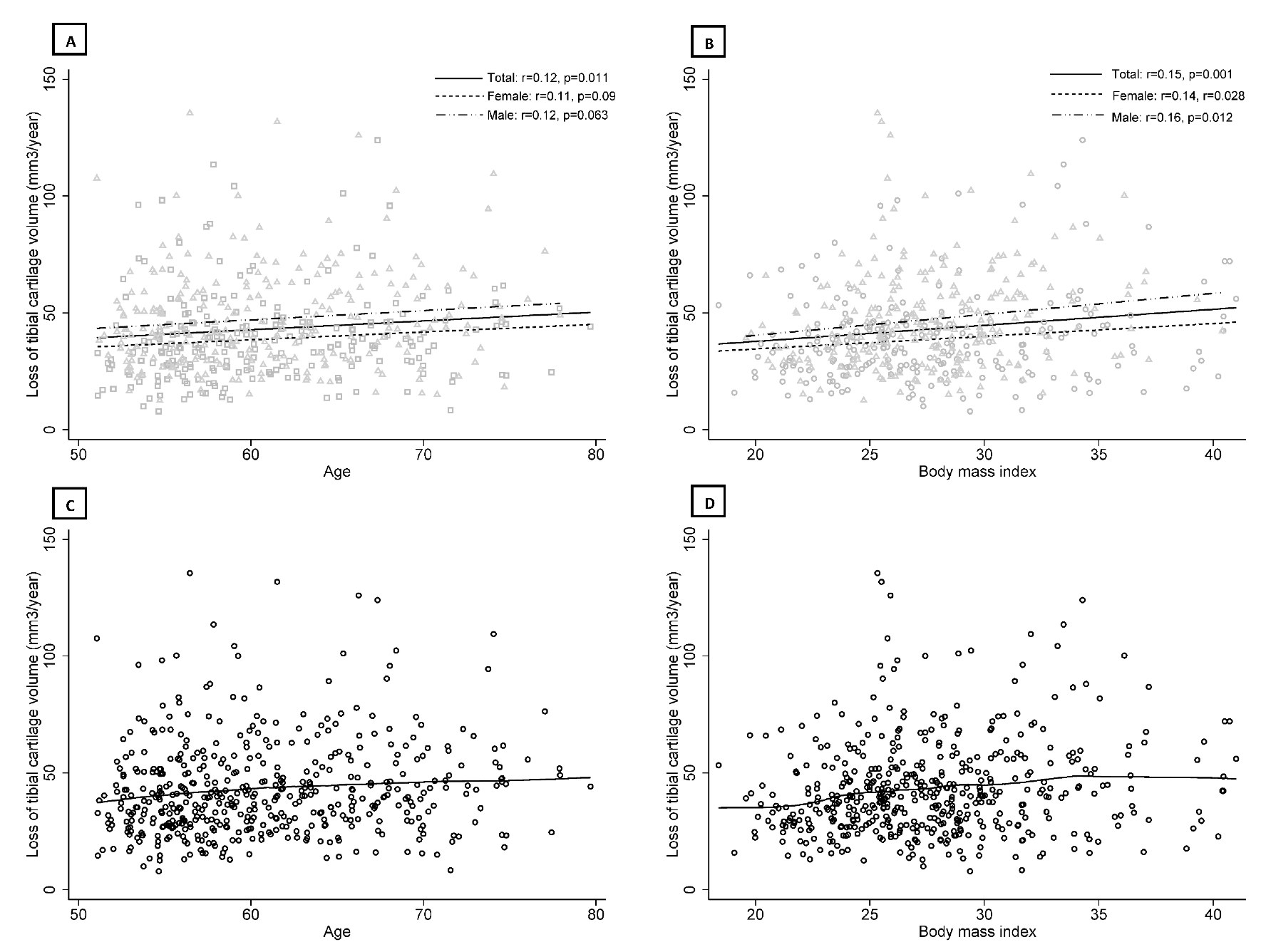
Figure S1.** Linear (A and B, triangle indicates males and circle females) and non-linear (C and D) associations of age and body mass index at baseline with loss of tibial cartilage volume (mm^3^/year).


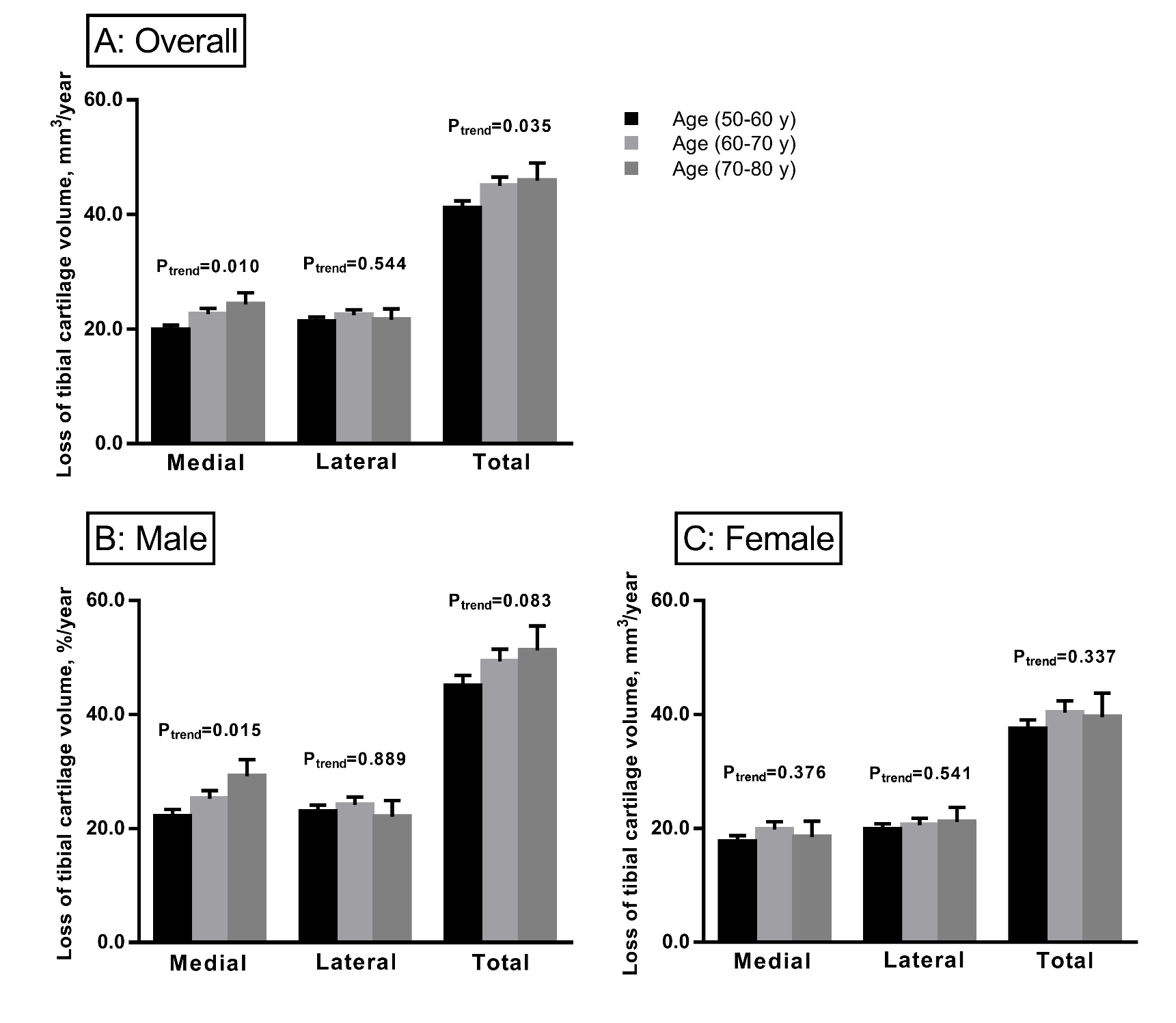
**Figure S2.** Loss of tibial cartilage volume among age groups over 10.7 years overall (A) and in males (B) and females (C). Bar graph indicates the mean value of tibial cartilage loss (mm^3^/year), and error bars indicate standard errors. *P* for trend was calculated by univariable linear regression models.


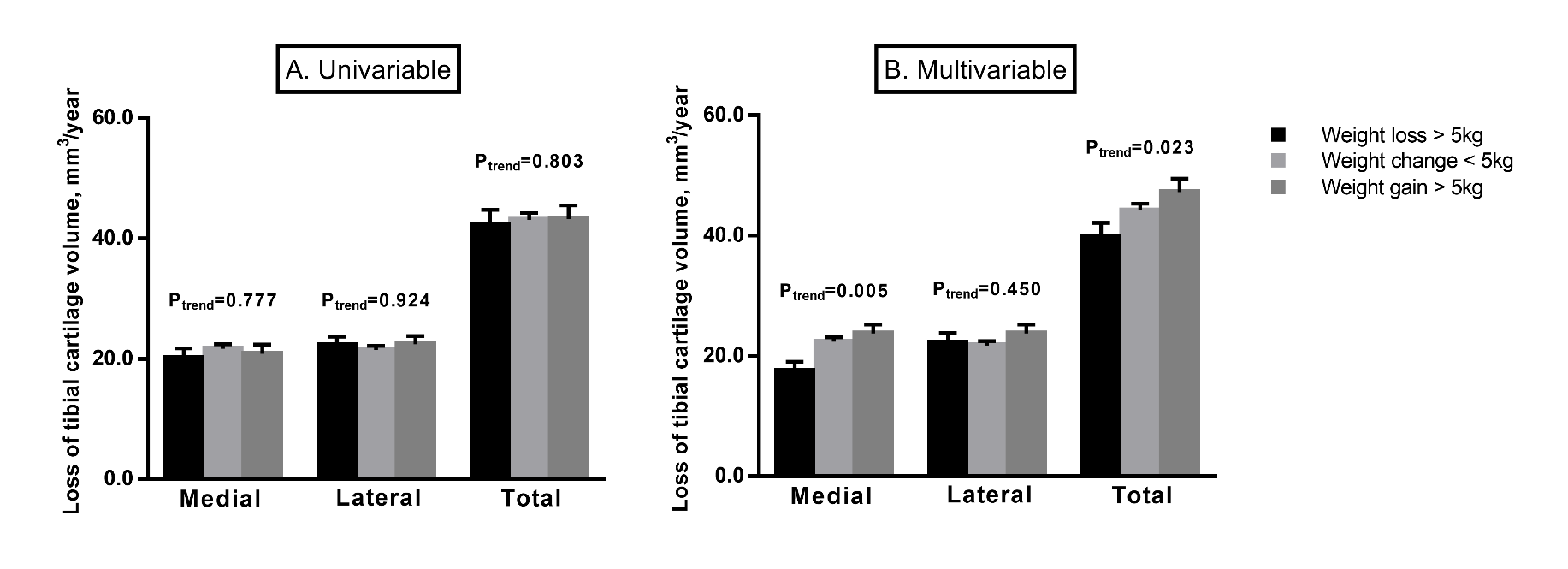
**Figure S3.** Uni- (A) and multivariable (B) analyses for the association between weight change and loss of tibial cartilage volume. Bar graph indicates the mean value of tibial cartilage loss (mm^3^/year), and error bars indicate standard errors. Multivariable analyses adjusted for age, sex, body mass index, radiographic osteoarthritis, history of knee surgery and knee injury, physical activity and site-specific tibial cartilage volume at baseline.
